# Diet in the Second Year of Life

**Published:** 1912-10-19

**Authors:** 


### MATERNITY AND MOTHERING.

(Inquiries and Correspondence are invited.)

### Diet in the Second Year of Life.

During the earliest weeks of a baby's existence there is generally little difficulty in getting those who have charge of it to listen to expert advice on the subject of how it should be fed. True, counsels of perfection are not always followed, and in too many cases are not possible. True also that some of the most ignorant mothers—of the "just what we 'as ourselves" type—are scornful of advice and cannot be dissuaded from gin, bananas, biscuits, and tea as ingredients of diet for their babies. But in the main the lump of maternal indiscretion is being slowly leavened by the teachings of the experienced regarding the feeding of nurslings.

### Dietary Mistakes after Dentition.

Much less attention, however, is paid to the inculcation of correct principles of diatetics for children in their second year. At that age babies can, no doubt, survive dietary mistakes which would speedily have serious consequences in the first year; their digestions are more elastic, so to speak. Advice is not thought necessary about so simple a matter as the nourishment fit for a child which has got teeth to chew with. Yet mistakes are easy to make, and very commonly indeed are made, even by those who take the utmost care over the feeding of their young infants. And the consequences of those mistakes, instead of being rapidly apparent as would be the case in early infancy, do not appear for months or years, and hence are not ascribed to their true cause.

In ideal circumstances a breast-fed baby should be weaned gradually during the eighth and ninth months of its life. That is, the process of weaning should be complete by the time the child is a full nine months old. In rare cases foolish mothers of the less educated classes continue to nurse their babies much longer than this, even up to two years in some cases. It is only necessary to state emphatically that this practice is fraught with grave risks of ill-health both to mother and child, and to condemn it out of hand.

With the details of weaning we are not concerned for the moment, though the process is one which is not always quite so perfectly simple as one might suppose. Let us assume that the nursling has been successfully weaned, and is taking a milk diet from a bottle without any difficulty; or that it has been weaned in the early stages of its existence and hand-fed with success thereafter. Let us assume also that the baby is well into its tenth month, and is healthy and thriving. How many meals should this child get, and what should they consist of? Most babies at this age do well on five meals a day: sometimes it is necessary to give six, and it is certainly most desirable that six should not be exceeded. In quantity each meal should consist of seven ounces of milk, with an ounce of water added if pure milk disagrees, two to four drachms of cream, and one to two drachms of sugar. Demerara sugar is perhaps the best to be recommended; there is no need at this age to use the more expensive milk-sugar (lactose). One or two of these meals may contain instead of cream a malted or cereal food of approved manufacture. The juice of a grape or two is also a valuable item in the day's dietary, especially if a tendency to constipation exists.

At a year old it is generally advisable to accustom the child to take two or three meals a day from a cup or spoon instead of from the bottle. Now is the time for additions to the diet, such as oatmeal, bread-crumbs, rusks soaked in milk, bread and milk, and mashed potato and gravy. Another important item of diet is something hard to chew at, not so much as a food as to help in developing the teeth and the jaws. A hard crust or a hard biscuit serves the purpose well, and this may be very thinly spread with butter if the child shows a disposition to reject it when offered plain. At this age of one year some doctors advise that the quantity of milk should be somewhat cut down, on the ground that too much of this satisfying food spoils the appetite for more varied fare. In this country, however, it is usual to allow one and a-half to two pints of milk a day; the former quantity is quite sufficient for most children.

### The Number of Meals and Variety

Exactly at what age the number of meals should be cut down from five to four authorities differ: some say at a year, others eighteen months, others two years. In the writer's opinion fifteen to eighteen months is the right time for this change to be introduced; and in most cases nearer to the earlier of these ages. At fifteen months, too, or even earlier, a soft boiled egg may be introduced into the diet-sheet. Green vegetables may be added to the potato and gravy; and broth or soup with bread soaked in it is useful as a change.

By eighteen months, or even sooner, rice, custard, sago, or other milk puddings may be given; bread and butter and jam are also appreciated. Stewed fruits, such as prunes or plums, are much enjoyed, and fresh fruit, such as bananas. Lean meat may now be added cautiously to the list: at first it must be minced, but this precaution should be abandoned as soon as possible, care being taken to see that the child has only small bites and masticates them thoroughly. Fresh boiled fish can also now be given. Water, or barley-water, should now be given with the midday meals, milk being reserved for breakfast, teatime, and bedtime. Tea should not be given at all during the second year of life. Sweets also should form no part of infant dietary. Fruit is far better for the child, and especially for his teeth; and if brought up to take it regularly, he will most likely never develop that excessive craving for sweets which characterises children who have been fed too much on sloppy foods.

